# Dynamics of Canadian perceptions of low-dose radiation following science-based educational communication sessions

**DOI:** 10.3389/fpubh.2026.1976699

**Published:** 2026-10-09

**Authors:** Francisco Javier Sahagún-Aguilar, Margot Hurlbert

**Affiliations:** Johnson Shoyama Graduate School of Public Policy, University of Regina, Regina, SK, Canada

**Keywords:** Canada, infographic-based communication, low-dose radiation, risk perception, science communication

## Abstract

This study examined whether participation in science-based Educational Communication Sessions (ECS) involving infographic-based materials, scientist-attributed information, and focus-group discussion was associated with changes in public perceptions of low-dose radiation (LDR). Surveys conducted in Alberta, Saskatchewan, and Ontario measured participants’ self-reported perceptions before and immediately after participation in ECS involving two infographic formats: one explaining LDR through everyday analogies and another describing safety protocols in hospitals and nuclear power plants. Binary logistic regression was used to explore associations between demographic characteristics and whether participants reported improved LDR perceptions following participation in the ECS. Participants generally reported more favorable perceptions of LDR and high levels of trust in scientists following participation in the ECS. Participants attending sessions involving the analogy-based infographic showed more consistent favorable shifts across provinces, whereas larger favorable shifts were observed in Saskatchewan and Ontario than in Alberta following sessions involving the safety-focused infographic. These findings suggest that science-based ECS incorporating infographic materials may be associated with immediate self-reported improvements in LDR perceptions. Given the exploratory design, the absence of a control group, the modest sample size, and the non-representative sample, the findings should be interpreted cautiously as preliminary evidence to inform future research on science communication and public perceptions of low-dose radiation.

## 1 Introduction

Trust and mistrust in science communication has implications for personal health decisions and broader public health policy as experienced and documented in the recent Coronavirus pandemic (1). Similar dynamics have existed in the context of Low Dose Radiation and associated medical interventions such as X-rays, mammograms etc. and technology including Small Modular Reactors (SMRs) (2–4). Trust in information sources may influence decisions concerning medical treatments (5) and public responses to emerging technologies such as SMRs (2).

Low-dose radiation is a particularly challenging topic for public risk communication because scientific assessments of its health effects involve uncertainty at low exposure levels (6, 7). Radiation protection has traditionally adopted conservative approaches to low-dose exposure, while scientific discussions continue regarding the magnitude and interpretation of risks at very low doses (7, 49). These differences do not necessarily represent disagreement about whether radiation can cause harm, but rather uncertainty concerning the shape and magnitude of the dose–response relationship at low exposure levels (ICRP 2005). Consequently, communicating LDR requires distinguishing between established radiation-protection principles, scientific uncertainty, and public perceptions of risk.

Risk perception, trust in scientists, and institutional trust represent related but distinct concepts. Risk perception refers to how individuals assess the potential likelihood and severity of a hazard. Trust in scientists concerns the credibility participants attribute to scientific experts as sources of information, whereas institutional trust concerns confidence in organizations responsible for regulating, managing, or delivering activities involving radiation. These concepts may be related, but they should not be treated as interchangeable. More generally, trust involves confidence in the reliability or safety of a person or thing (46).

Previous research has documented public perceptions of low-dose radiation, trust in scientific and institutional actors, and concerns surrounding nuclear technologies. However, less is known about how participants’ self-reported perceptions of LDR may change immediately following structured, science-based communication activities using different communication formats. Examining these responses across different provincial contexts may provide preliminary insights into how participants respond to different approaches to science-based LDR communication.

This research examined participants’ self-reported perceptions of low-dose radiation before and after participation in science-based ECS involving two infographic formats. The study also examined participants’ trust in scientists and explored whether demographic characteristics were associated with participants reporting improved LDR perceptions following participation in the Educational Communication Sessions.

The research answers the question, “How do participants’ self-reported perceptions of low-dose radiation differ before and after participation in science-based Educational Communication Sessions delivered through focus groups using two infographic formats in Alberta, Saskatchewan, and Ontario?” To address this question, we employed an exploratory mixed-methods design in which participants completed surveys before and after participation in science-based Educational Communication Sessions presented within province-specific focus groups.

## 2 Materials and methods

This study was conducted between October and December 2023, which places it in a post-pandemic context. This timing is relevant when interpreting results, as levels of institutional trust in Canada—like in many countries—shifted substantially following the COVID-19 pandemic.

### 2.1 Study design

This study employed an exploratory single-group pre-test-post-test mixed-methods design. Participants completed a survey before exposure to infographic materials and a second survey immediately following exposure. The design was intended to assess changes in participant perceptions associated with the communication intervention. Because no separate control group was included, the study was not designed to support strong causal inference comparable to randomized controlled trials. Instead, participants served as their own baseline through pre-intervention measurement. The study was not preregistered and should be understood as exploratory rather than confirmatory. This study examines public responses to different educational communication materials derived from scientific and institutional sources only.

Focus groups were scheduled and organized in Saskatchewan, Ontario, and Alberta in proportions according to each province. Insightrix, an experienced firm hired to avoid any potential bias between the researcher and participants, conducted the recruitment. They used demographic stratification from their database (age, gender, education, and household income) to approximate provincial diversity within the constraints of a qualitative study. Participants were not required to have prior knowledge of radiation science; however, Table 1 includes their self-reported perceptions of their radiation knowledge prior to viewing the infographics (Pre-Infographic). The research occurred in three steps:

**Table 1:** Changes in participants’ self-reported perceptions of LDR following the ECS involving infographic 1.

Infographic and province	Moved from neutral to positive	Stayed positive	Moved from negative to neutral or positive	Moved from positive to neutral	Moved from positive to negative
Infographic 1 in Saskatchewan	40%	85%	67%	15%	0%
Infographic 1 in Alberta	67%	86%	67%	14%	0%
Infographic 1 in Ontario	50%	75%	67%	17%	8%

*Step 1*: Survey pre-infographic. The participants were asked about their demographic data (type of infographic, gender, age, income, and academic level) and perceptions about LDR in a Likert scale from 1 to 5.

*Step 2*: Access to infographics within focus groups. Participants accessed the infographics in focus groups organized by province (Alberta, Saskatchewan, and Ontario). Each province included several focus groups that reviewed either Infographic 1 (everyday low-dose radiation analogies) or Infographic 2 (protocols and protection rules in medical and nuclear settings). The specific number of participants per group and infographic is detailed in Table 2, “Distribution of focus groups by province and infographic.” Infographic 1 is in Appendix A, and infographic 2 is in Appendix B. Participants were informed that the research was being conducted by the University, and a scientist presented a taped ten-minute discussion of the infographic assigned to each focus group.

**Table 2:** Distribution of focus groups by province and infographic.

Province	Total participants	Infographic	Focus groups included	Participants per group
Saskatchewan (SK)	46	1	1, 2, 3	6, 7, 8
		2	4, 5, 6	10, 9, 6
Alberta (AB)	54	1	7, 8, 9	9, 9, 8
		2	10, 11, 12	9, 10, 9
Ontario (ON)	50	1	13, 14, 15	7, 9, 8
		2	16, 17, 18	9, 10, 7

*Step 3*: Post-infographic survey. The people answered a second survey about their perceptions of LDR after accessing the infographics.

#### 2.1.1 Survey and focus-group instrument

The complete survey instrument and focus-group questions are provided in Appendix D. The instrument included demographic and background questions, pre and post-session measures of LDR perception, questions concerning participants’ responses to the Educational Communication Session, and exit questions assessing perceptions of LDR and its benefits and risks. The focus-group questions also included open-ended questions designed to explore participants’ prior understanding, concerns, perceived benefits, and responses to the information presented during the session.

For clarity, the combination of infographic exposure, scientist-attributed information, and focus-group participation is referred to throughout this manuscript as the Educational Communication Session (ECS). This term is used to describe the overall communication context experienced by participants rather than the infographic materials alone.

While this research employs a mixed-methods approach consisting of both survey results and a qualitative research analysis of focus group discussions, it was not intended to be statistically representative since this is a common limitation of the focus groups method due to the high cost. Instead, it provides exploratory evidence regarding participants’ self-reported changes in LDR perceptions following standardized Educational Communication Sessions. Funding limitations and the time-intensive nature of conducting individual focus groups constrained the sample size; however, the data still offer meaningful preliminary patterns that motivate further research.

#### 2.1.2 Qualitative analysis

The qualitative component was used primarily to complement and contextualize the quantitative findings. Open-ended responses and focus-group comments were reviewed descriptively. For the specific analysis of participants’ trust in scientists, responses were additionally reviewed and classified into five categories: positive, neutral, negative, empty/unclassifiable, and atypical, with atypical responses referring to comments unrelated to trust in scientists. The researcher conducted this classification because responses to the trust-in-scientists question had not been previously categorized. Although efforts were made to apply the categories consistently and objectively, some degree of interpretive subjectivity cannot be excluded. These classifications were therefore treated as descriptive contextual information rather than definitive measures of participants’ trust in scientists.

#### 2.1.3 Quantitative outcome variable

The dependent variable for the binary logistic regression represented whether participants reported an improved LDR perception following participation in the Educational Communication Session. The original LDR perception scale ranged from 1 to 5, where 1 represented “safe,” 3 represented “neutral,” and 5 represented “dangerous.” For the purpose of the regression analysis, the pre and post-session LDR perception scores were reverse-coded so that higher values represented more favorable perceptions of LDR. Participants were then coded as 1 when their post-session score was higher than their corresponding pre-session score, and 0 otherwise, using the following algorithm:


$$\begin{array}{l} \text{Improved}\;\mathrm{LDR}\;\text{perception}\\ =\{\begin{array}{cc} 1, & \text{if}\;\mathrm{LDR}\;{\text{perception}}_{\text{post}}>\mathrm{LDR}\;{\text{perception}}_{\mathrm{pre}}\\ 0, & \text{if}\;\mathrm{LDR}\;{\text{perception}}_{\text{post}}\leq \mathrm{LDR}\;{\text{perception}}_{\mathrm{pre}.} \end{array} \end{array}$$

This binary operationalization was used to examine factors associated with an increase in LDR perception following exposure to the infographic and to facilitate exploratory binary logistic regression modelling. Because dichotomization does not capture the magnitude of change in the original 1–5 scale, alternative operationalizations may yield different results. The pre and post-session LDR perception measures corresponded to Questions 1 and 12 of the instrument, respectively (Appendix D).

Alternative operationalizations and analytical approaches may yield different quantitative results. Future studies should therefore evaluate the robustness of these findings using alternative coding schemes, ordinal regression, mixed-effects models, or validated multi-item scales.

From a methodological perspective, the communication intervention was designed to be symmetric across study conditions. Within each province, participants were exposed to standardized infographic-based content, presented in the same format, duration, and informational structure, and supported by identical scientific references and institutional attribution. Within each province, the communication sessions followed a standardized format in terms of duration, presentation structure, scientific references, and institutional attribution. This standardization helped maintain consistency in how the two infographic-based communication approaches were presented across provinces.

The purpose of the study was to explore whether participants’ self-reported perceptions differed before and after participation in the ECS concerning low-dose radiation. While changes observed between pre and post-intervention measurements may be associated with participation in the ECS, alternative explanations such as repeated measurement effects, focus-group dynamics, social desirability effects, and participant interactions cannot be excluded.

#### 2.1.4 Design and expert validation of infographics

Two infographics were designed to convey key concepts related to LDR and its relevance to public support for SMRs (48). The first infographic used natural analogies to illustrate LDR exposure, including common foods and environments with quantifiable radiation levels, whereas the second infographic emphasized radiation protection measures implemented in hospital settings and nuclear power facilities.

*Infographic 1*: For natural radiation analogies and LDR dose comparisons, we used information, values, and examples from the following sources:

- Radiation Meaning (8);
- Radiation Levels (9);
- Radioactive Discharges (10);
- Canadian Dose Calculator (11, 12);
- World Nuclear Association’s Radiation and Health Effects (13);
- Smoking radiation exposure (14).

*Infographic 2*: Focused on protection protocols, and rules, besides Radiation Meaning (8) and Radiation Levels (9), sources also included:

- Imaging Fundamentals from the University of Saskatchewan [Brent (15)];
- Stanford Environmental Health and Safety’s Radiation Protection Guidance for Hospital Staff [Stanford Environmental (16)];
- Protecting Workers (17);
- Spent Fuel Management Options (18);
- Nuclear Energy Agency’s Radiation Transport and Shielding (19).

Pilot versions of both infographics were evaluated by members of the Canadian Nuclear Society (CNS). Their expert feedback informed revisions to the content and language to enhance scientific accuracy and clarity of communication. The revised infographics received final approval from CNS experts before being deployed in the study. Participants who accessed the infographics were informed of the institutions responsible for the materials. All procedures complied with the requirements established by the University of Regina’s Research Ethics Board.

## 3 Results

### 3.1 People’s LDR perceptions before and after the study

Previous Canadian research has documented relatively low levels of public trust in nuclear and radiation-related institutions. For instance, studies in Saskatchewan (1,104 participants) and Ontario (1,008 participants), have found that over 70% of the individuals perceive even very small radiation doses as dangerous, reflecting elevated risk sensitivity and limited confidence (20). Similarly, a 2008 provincial survey in Saskatchewan (21) reported that 84% of respondents opposed the construction of a nuclear power plant, and the people expressed a preference for other sources of energy [C. B. C. (22)] a result that contributed to the cancellation of a major proposed facility (23). these findings are consistent with well-established historical shifts in public trust following major nuclear accidents such as three Mile Island and Chernobyl, which substantially altered risk perceptions internationally (24, 25).

In contrast, participants in our focus groups reported comparatively more favorable perceptions of LDR. Because our sample is not statistically representative, these results should be interpreted as exploratory and limited to the participants studied. Differences between studies may reflect differences in sampling, study design, question wording, and study context. Further research using comparable designs would be needed to examine whether these patterns are consistent across populations.

Previous Canadian surveys have reported varying levels of trust in government as a source of information about LDR. For instance, Hurlbert et al. (20) found that 65% of respondents expressed low trust in government communication on LDR, while an OECD report (26) noted that 49% of Canadians expressed trust or moderately high trust in the federal government. These differences likely reflect variations in survey design, sampling frames, and question wording rather than direct shifts in public trust over time. For this reason, these findings are used here for contextual background rather than for direct comparison.

Recent national surveys also show that trust in Canadian institutions fluctuated substantially during and after the COVID-19 pandemic (45). For instance, the Edelman Trust Barometer reported that trust in the federal government rose sharply from 50% in late 2019 to 70% in early 2020, before declining again as the pandemic progressed. Similar volatility was observed across other institutions (27, 28).

### 3.2 Participant’s trust in Scientists

The following table was created to display the analysis of qualitative responses in relation to trust in scientists. Qualitative answers were translated into numerical values to categorize the data when participants were asked about their trust in scientists after viewing the infographics. Comments were categorized (Table 3) as positive, neutral, negative, or unclassifiable (empty spaces) or atypical (referring to another topic).

**Table 3:** Classification of comments about trust in scientists according to the infographic accessed by the respondents.

Data type	Trust in scientists			
	Infographic 1	Infographic 1 (%)	Infographic 2	Infographic 2 (%)
Sample size	71	100%	79	100%
Positive comments	48	68%	56	71%
Neutral comments	9	13%	10	13%
Negative comments	5	7%	8	10%
Empty spaces	6	8%	2	3%
Atypical	3	4%	3	4%

After presentation of both infographics there was a demonstrated similar positivity in the perceptions of participants about scientists as expressed in their verbal comments. At the beginning all the participants were informed that the university was behind this research and LDR scientists had helped in the preparation of the infographics. This contextual information may help explain why participants reported positive perceptions of scientists following the ECS. However, the study design does not permit identification of which specific components of the session contributed to these responses.

These findings are consistent with the literature. Greenberg’s (29) research in the United States of America found independent scientists and regulatory bodies were regarded as the most trustworthy sources of information. Similarly in Canada, scientists in one study were the group with more support and trust to communicate about LDR; only 6.5% of survey respondents did not trust them, followed by the Canadian nuclear regulator with only 11% expressing a lack of trust (20).

### 3.3 Change in perceptions after infographic—provincial comparison

Although Figure 1 indicates that participants reported generally more favorable perceptions following the ECS in all three provinces, these results must be interpreted as exploratory due to the small and non-representative provincial subsamples (common limitation from the focus groups method due to the high cost). Figure 1 presents participants’ average self-reported perceptions of LDR before and after exposure to the science-based educational materials in each province and infographic condition.

**Figure 1:**
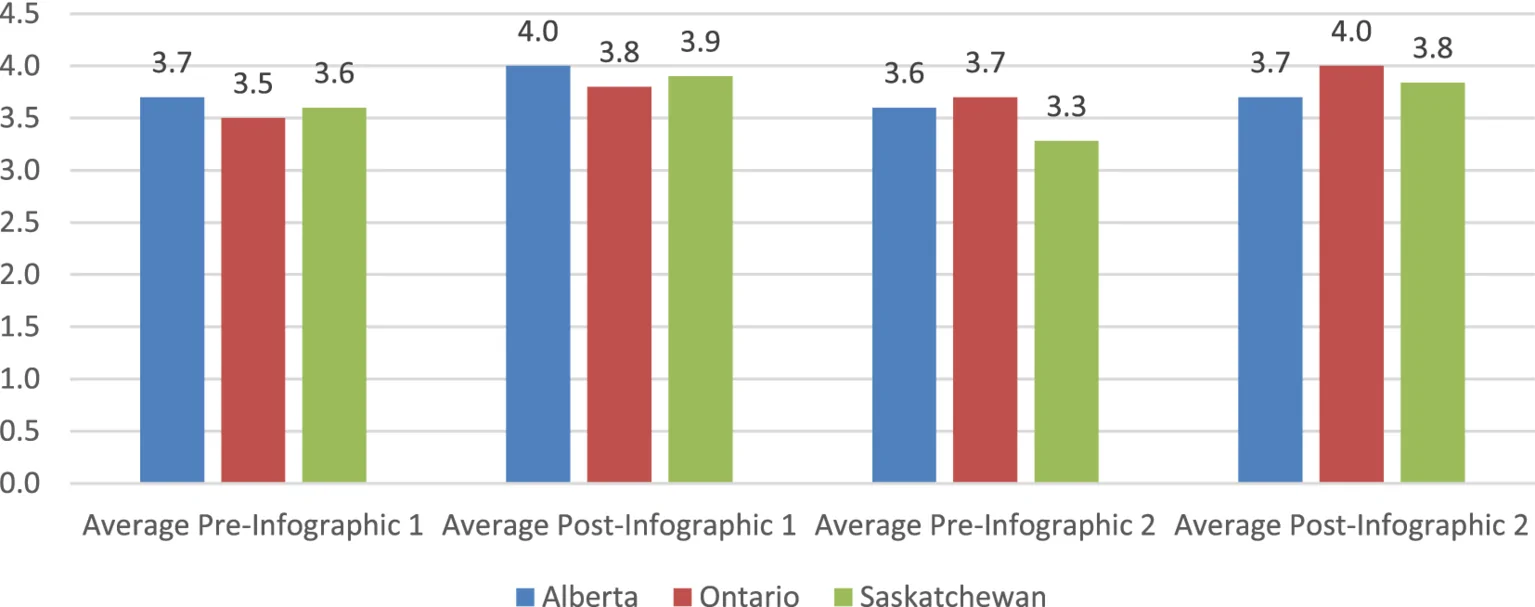
Average self-reported perceptions of LDR before and after exposure.

Average participant self-reported perceptions of LDR before and after exposure to the science-based educational materials, by province and infographic type. Survey responses were originally collected using a 1–5 Likert scale, where 1 = safe, 3 = neutral, and 5 = dangerous. For graphical presentation, responses were reverse-coded (1 → 5, 2 → 4, 3 → 3, 4 → 2, and 5 → 1), so that higher values represented more favorable perceptions of LDR. All analyses used the original 1–5 response scale.

To provide additional context, Table 4 summarizes participants’ self-reported prior knowledge about radiation: in Saskatchewan (*n* = 46), 41.3% reported Moderate and 32.6% Poor knowledge; in Alberta (*n* = 54), 37% reported Moderate and 31.5% Good knowledge; and in Ontario (*n* = 50), 38% reported Moderate and 28% Poor knowledge. These patterns indicate that participants across provinces entered the study with low-to-moderate familiarity with radiation topics.

**Table 4:** Participants’ self-reported prior knowledge about radiation by province.

Knowledge level	Saskatchewan (*n* = 46)	% (SK)	Alberta (*n* = 54)	% (AB)	Ontario (*n* = 50)	% (ON)
Very good	0	0.0%	2	3.7%	4	8.0%
Good	7	15.2%	17	31.5%	9	18.0%
Moderate	19	41.3%	20	37.0%	19	38.0%
Poor	15	32.6%	12	22.2%	14	28.0%
Very poor	2	4.3%	0	0.0%	2	4.0%
Do not know/no	3	6.5%	3	5.6%	2	4.0%
Total	**46**	**100%**	**54**	**100%**	**50**	**100%**

Bold values indicate the total number of participants in each province.

Insightrix, the professional research firm contracted for recruitment, selected participants to approximate provincial distributions in key demographics—including age, gender, education, and household income—yet the resulting subsamples remain too small to draw population-level conclusions. Consequently, differences such as participants exposed to Infographic 2 during the ECS reported more favorable responses in Saskatchewan than in Alberta within this sample should be regarded as preliminary observations within this sample, not as evidence of broader provincial differences. Larger, statistically representative samples would be required to examine these patterns with confidence. Alberta and Saskatchewan differ in population size, economic structure, and levels of industrial activity, which may provide contextual clues—but not causal explanations—for the different response patterns observed between provinces. Alberta has approximately four times Saskatchewan’s population (4.88 million vs. 1.24 million in 2024) and a significantly larger GDP ($344.1B vs. $77.9B in 2023), reflecting a more industrialized economy (30, 31); (32, 33). Alberta’s economy is led by oil and gas, followed by agriculture, whereas in Saskatchewan agriculture is the primary sector. Prior research suggests that communities embedded in more industrialized contexts may have different baseline experiences with regulation, risk communication, and technical institutions. While our study cannot establish causality, these structural differences may help contextualize why Infographic 2—which focuses on protocols and rules—resonated differently across the two provinces.

These contextual differences help explain why natural gas power production tends to be viewed more favourably in Alberta compared to other Canadian provinces. Two-thirds (67%) of Canadians support building new natural gas generation capacity, but support is highest in Alberta (78%) (34). This is significantly above the national average, likely reflecting the province’s long-standing economic connection to oil and natural gas.

This might help contextualize why participants exposed to Infographic 2 reported relatively favorable responses in Alberta, although it was the lowest among the three provinces. One possible interpretation—based on participants’ comments and contextual differences between provinces—is that residents of Alberta are more accustomed to a highly regulated oil and gas environment. Because exposure to safety rules is part of their daily experience, information framed around regulatory content may feel less novel or impactful to them. In contrast, this may help clarify why participants in Saskatchewan showed a stronger reaction to infographic 2, as this kind of regulatory messaging might be less common in their daily context. However, given the small sample size, these interpretations should be considered tentative.

Ontario is a much bigger province in all aspects, having a population of 15,996,989 on April 1, 2024 (35), and a GDP of $887.7 billion (36). This is nearly 12 times larger than Saskatchewan and almost 3 times larger than Alberta. Therefore, we could expect that the effect from infographic 2 would be similar to that of Alberta. However, it was in the middle of both provinces with more favorable responses than those observed in Alberta, but lower than Saskatchewan. This higher positivity in Ontario than Alberta could also be explained due to the fact that Ontario is a province that has had nuclear industry since 1962, and this information may not be new to Ontario residents.

### 3.4 Changes in LDR risk perception following each infographic

The question “On a scale from 1 to 5, what is your perception when you hear the term low dose radiation?” was asked after a brief preamble in which the moderator explained that the focus group would discuss LDR in relation to human health and everyday exposures.

Therefore, although the wording of the Likert-scale prompt is concise, participants answered it within a clearly defined context: they understood that the question referred to perceived risk to humans associated with LDR exposure. This clarification strengthens the interpretation of the scale, in which 1–2 indicate lower perceived risk, 3 represents a neutral position, and 4–5 reflect higher perceived risk.

Figure 2 provides the data collected pre infographics in each province.

**Figure 2:**
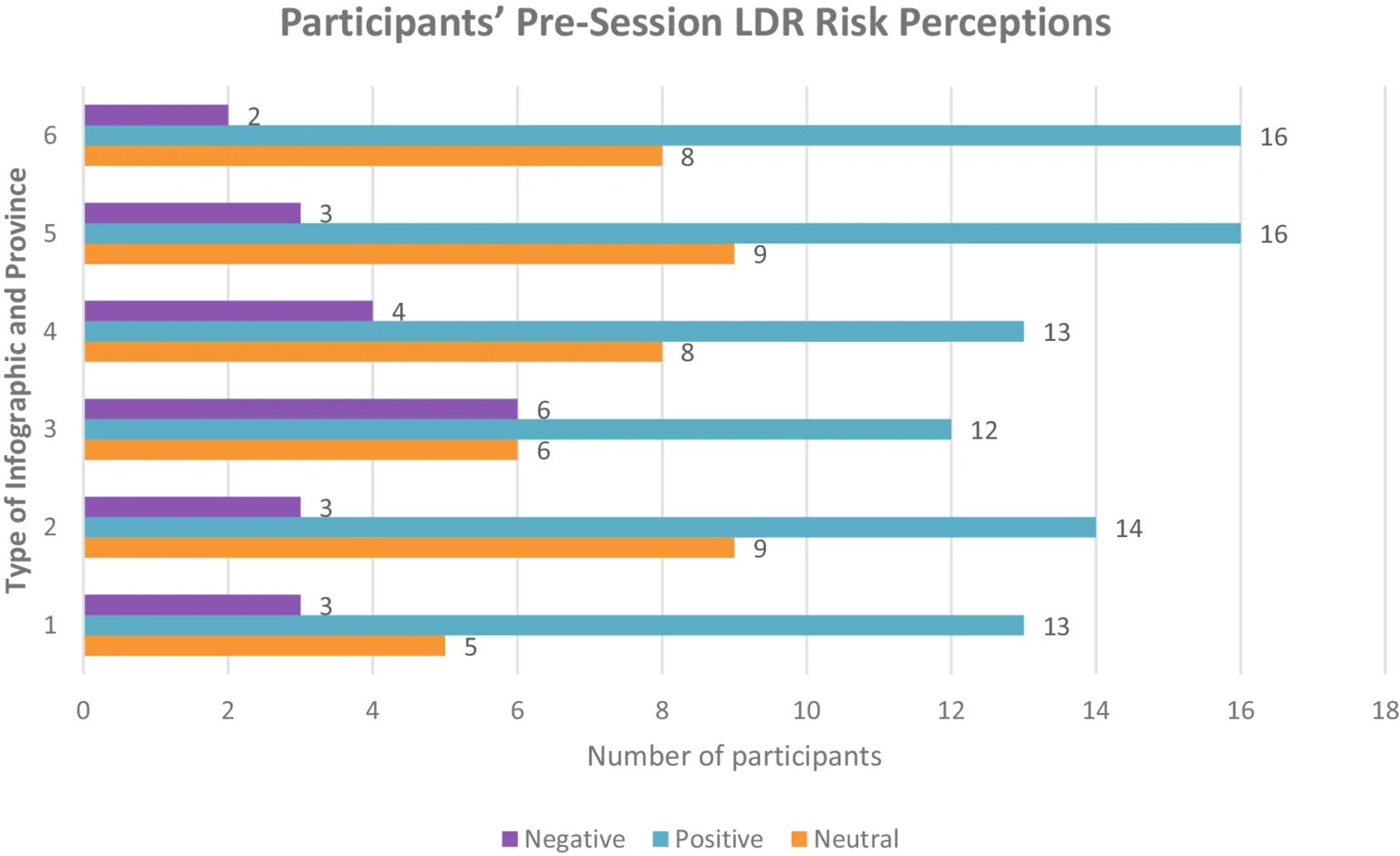
Pre-session LDR risk perceptions by province and infographic type.

Table 1 then shows changes in participants’ LDR risk perceptions following Infographic 1, while Table 5 shows changes following Infographic 2.

**Table 5:** Changes in participants’ self-reported perceptions of LDR following the ECS involving infographic 2.

Infographic and province	Moved from neutral to positive	Stayed positive	Moved from negative to neutral or positive	Moved from positive to neutral	Moved from positive to negative
Infographic 2 in SK	50%	77%	75%	15%	8%
Infographic 2 in AB	56%	69%	33%	25%	6%
Infographic 2 in ON	50%	88%	100%	13%	0%

In general, participants reported more favorable perceptions following the ECS involving Infographic 1. The negative qualitative comments were limited in number and fell mainly into two themes: (1) a desire for more detailed or technical information, and (2) concerns that simplified analogies might omit scientific nuance. These concerns were raised by a small minority of participants—between 3 and 6 individuals per province (representing 11–25% of provincial subsamples)—and did not indicate perceptions of bias.

In focus group settings, such comments commonly reflect requests for elaboration rather than distrust. The vast majority of participants expressed that the infographic was clear and informative, and these concerns did not substantially alter the overall pattern of more favorable self-reported perceptions observed following the ECS across the sample.

In research in the United States, in which analogies were not regarded as one of the most successful methods to communicate LDR, the research company, Bisconti, suggests that this happens because analogies are not addressing the aspect of protection (36). This suggestion potentially could explain part of the information issue in infographic 1, and uncertainty about why some participants reported less favorable perceptions in the post-session survey. Future research should consider how to fulfill this need of information from a small number of people post infographic presentation.

Similarly, the majority of participants had relatively favorable LDR risk perceptions in all three provinces before Infographic 2, as depicted in Figure 2. Table 5 displays changes in participants’ LDR risk perceptions following Infographic 2.

In general, participants reported more favorable perceptions in the post-session survey than in the pre-session survey. However, again any negative comments about this infographic were oriented to asking for clearer information about LDR and how safety rules specifically protected workers in nuclear power plants. Participants said the information about hospitals was clear already. These negative comments asking for clearer information about LDR and workers protection in nuclear power plants may help contextualize why some participants reported less favorable perceptions in the post-session survey. However, when this occurred, most participants tended to move toward a neutral perception rather than a more negative perception.

According to previous research people have a bigger tendency to “disagree” with something complicated than saying “I don’t understand” (37). Also, one research study suggested that a big cognitive load and complexity of a subject play an important role in negative perception. Roberts (38) suggest that people tend to reject what they do not understand due to many different reasons including fear of the unknown, fear of change because the new knowledge may suggest that a new change is necessary, and also a perception of danger in relation to what we cannot understand (39).

Limited time to communicate with people, grab their attention, and observe any shifts in perception makes addressing these potential explanations of a negative response challenging. Future research could consider modifications to infographics to provide for further information in the form of questions and answers, or websites that can be accessed for more information. Potentially opportunity to participate in future research might also be a potential solution.

### 3.5 Improved LDR perceptions following the educational communication session

In general, participants reported favourable responses following the ECS involving both infographic formats.

While descriptive patterns by gender, age, education, and income were examined, the small sample sizes within each subgroup do not allow statistical inference. Therefore, these demographic differences are presented as exploratory observations only, and should not be interpreted as statistically meaningful.

The following section first reports the statistical significance of the overall provincial results (and combined results), followed by a descriptive summary of any observable patterns. Statistical tests were conducted using a significance threshold of 0.05.

Given the modest sample size (*n* = 150 across provinces), results should be interpreted cautiously, as the study was not powered to detect small effects.

Therefore, statistical outcomes are reported strictly in terms of whether they reached the 0.05 threshold, without interpreting marginal or near-significant findings. The primary value of the statistical analysis in this study is descriptive and exploratory, providing preliminary indications that may guide future, larger-scale research. Using the conventional significance threshold of 0.05, only the Alberta logistic regression model reached statistical significance based on the Omnibus Tests of Model Coefficients (Saskatchewan *p* = 0.362; Alberta *p* = 0.040; Ontario *p* = 0.706).

A regression analysis combining data from the three provinces also did not yield statistically significant results (*p* = 0.243).

Therefore, although the Alberta model reached statistical significance, the combined provincial analysis did not. Given the exploratory design, modest sample sizes, and inconsistent findings across provinces, these results should be interpreted cautiously and considered preliminary.

The SPSS Binary Logistic Regression analysis was performed to examine the association between the independent variables and the improved LDR perception. Independent variables were included to examine whether higher or lower estimated odds of the dependent variable were associated with specific participant characteristics or infographic conditions. The independent or predictor variables for this regression were the type of infographic (infographic one versus two), gender, age, income and academic background.

Detailed logistic regression results for each province are presented in Appendix C. Differences in the estimated associations between demographic variables and improved LDR perceptions were observed across provinces. Table 6 contains the coding of each variable, and Table 7 summarizes the logistic regression results.

**Table 6:** Coding of independent variables used in the binary logistic regression.

Coding for independent variables	
Infographic	Infographic 1 = 1 (the reference value).
Gender	Female = 1 (the reference value). Male = 2.
Age	18 to 24 = 1, 25 to 34 = 2, 35 to 44 = 3, 45 to 54 = 4, 55 to 64 = 5, (65 or more) = 6.
Education	Some high school = 1, Completed high school = 2, Some technical school or college = 3, Some university = 4, Completed technical or college diploma = 5, undergraduate, master’s degree or PhD = 6.
Household income	less than $30,000/year = 1, $30,000 to $60,000 = 2, $60,000 to $90,000 = 3, $90,000 to $120,000 = 4, $120,000 to $150,000 = 5, $150,000 or more = 6.

**Table 7:** Summary of results.

Type of analysis	Conclusions
Section 1, Participants’ LDR Perceptions Prior to the ECS	Within this exploratory sample, participants generally reported relatively favorable baseline perceptions of LDR prior to participation in the Educational Communication Sessions. Given the modest, non-representative sample, these findings should not be generalized to the broader Canadian population. Future research using larger and more representative samples could examine baseline LDR perceptions across different provincial and national contexts.
Section 2, Participant’s Trust in Scientists	Across all the ECS involving Infographic 1, 68% of participants expressed positive trust in scientists. Following all the ECS involving Infographic 2, 71% expressed positive trust in scientists. Because participants were informed that the educational materials were developed with scientific and institutional sources and were introduced to the scientists involved in the research, the study cannot determine the extent to which these responses were associated with any specific component of the ECS.
Section 3. Changes in Participants’ Self-Reported Perceptions Following the ECS	Regardless of participants’ baseline perceptions (negative, neutral, or positive), the overall descriptive tendency was toward more favorable self-reported perceptions of LDR following the ECS. Given the exploratory design, the absence of a control group, and the non-representative sample, these findings should be interpreted as immediate self-reported shifts rather than evidence of causal effects.
Section 4. Changes in LDR Perceptions by Infographic Format within ECS	ECS involving both infographic formats were generally associated with maintenance of positive perceptions or shifts toward more favorable perceptions of LDR among many participants. A smaller proportion of participants reported shifts from positive to neutral or negative perceptions, suggesting that ECS may be interpreted differently across individuals. Because the study design does not isolate the contribution of individual components of the ECS, these findings should be interpreted descriptively rather than causally.
Section 5, Exploratory Demographic Associations with Improved LDR Perceptions Following the Educational Communication Sessions	Only the Alberta provincial logistic regression model reached conventional statistical significance at the model level (Omnibus *p* = 0.040), whereas the Saskatchewan, Ontario, and combined models did not. At the individual predictor level, only age in the combined provincial analysis reached statistical significance (OR = 0.781, *p* = 0.043). None of the individual demographic predictors reached statistical significance within the provincial models. Directional tendencies observed for gender, education, household income, and infographic type should therefore be interpreted as exploratory rather than confirmatory.

#### 3.5.1 Gender relationship

The Alberta logistic regression model reached statistical significance (Omnibus *p* = 0.040). However, none of the individual demographic predictors, including gender (OR = 2.891, *p* = 0.110), reached statistical significance. Gender nevertheless showed the largest estimated association within the Alberta model and may represent a directional tendency warranting further investigation in larger samples.

Descriptive patterns were also observed across provinces. In Saskatchewan, women appeared to respond more favorably to Infographic 2 than to Infographic 1 regarding the perception that LDR is well managed. In Alberta, men appeared to respond more favorably to Infographic 1 than women, consistent with the direction of the gender coefficient observed in the Alberta model. These province-specific tendencies suggest that gender may interact with infographic style in different ways across regions. However, because the study was not designed or powered to test demographic subgroup effects, these observations should be interpreted as exploratory and warrant further investigation in larger samples.

Surveys conducted in Japan following the Fukushima disaster also revealed a notable gender difference in concerns. Among those surveyed, 71% of women and 52% of men were concerned about food safety (sample size = 1,167) (40). Additionally, 57% of women and 48% of men were highly cautious about their meals, and 35% of women compared to 23% of men reduced their time spent outdoors (sample size = 867) (47).

Previous literature has reported gender differences in risk perception and responses to environmental and health hazards. Although gender showed the largest estimated association within the Alberta model (OR = 2.891), the coefficient did not reach statistical significance (*p* = 0.110). Consequently, the present data do not provide statistical evidence of gender differences, and any observed patterns should be interpreted as exploratory.

#### 3.5.2 Age

Although the overall Saskatchewan model did not reach conventional statistical significance (Omnibus *p* = 0.362), the age coefficient within the model did not reach statistical significance (*p* = 0.084). In Saskatchewan, increasing age was associated with lower odds of reporting improved LDR perceptions. Given the exploratory nature of the study and the modest sample size, this finding should be interpreted cautiously and considered preliminary rather than confirmatory.

In the case of Saskatchewan for instance, the older the people are (each time the age increase per unit in the code), the odds decrease in 44.5% for the variable “Improved LDR perception”. In the case of Ontario, the tendency is negative similar to Saskatchewan, age decrease the odds 12.7% for the variable “improved LDR perceptions” per age unit increase, however, this result is far from being statistically significant. Previous literature has reported associations between age and attitudes toward risk, technology, and social change (41). However, the present study was not designed to identify the mechanisms underlying the observed patterns. Additionally, numerous studies suggest that aging is often associated with an increased tendency toward conservatism (42–44). Similarly, in Alberta, increasing age was associated with an estimated 19.9% decrease in the odds of reporting improved LDR perceptions per age category.

Across all three provinces, increasing age was associated with lower estimated odds of reporting improved LDR perceptions. This tendency was strongest in Saskatchewan (OR = 0.555), followed by Alberta (OR = 0.801) and Ontario (OR = 0.873). In the combined provincial analysis, increasing age was also associated with lower estimated odds of reporting improved LDR perceptions (OR = 0.781, *p* = 0.043), corresponding to an estimated 21.9% decrease in the odds per one-category increase in age. However, the overall combined regression model did not reach conventional statistical significance (Omnibus *p* = 0.243).

These directional tendencies should be interpreted cautiously and primarily as exploratory observations. Although age was not statistically significant within any individual provincial model, the age coefficient in the combined provincial analysis reached statistical significance (OR = 0.781, *p* = 0.043). Because the overall combined model was not statistically significant (Omnibus *p* = 0.243), this finding should be considered preliminary rather than confirmatory and warrants replication in larger samples.

No firm conclusions can be drawn regarding the mechanisms underlying these age-related patterns. Future studies with larger and more representative samples would be needed to determine whether these observed associations are robust and generalizable.

#### 3.5.3 Education

The association between education and improved LDR perceptions differed across provinces. In Saskatchewan and Alberta, higher education levels were associated with lower estimated odds of reporting improved LDR perceptions (OR = 0.880 and OR = 0.941, respectively), corresponding to modest decreases of approximately 12.0 and 5.9% in the estimated odds for each increase in education category. In contrast, Ontario showed the opposite directional tendency, with higher education levels associated with greater estimated odds of reporting improved LDR perceptions (OR = 1.219), corresponding to an estimated 21.9% increase in the odds per education category.

None of these associations reached statistical significance. Therefore, these provincial differences should be interpreted as exploratory directional tendencies rather than evidence of true educational effects. Future studies with larger samples are needed to determine whether these patterns are robust and reproducible.

#### 3.5.4 Infographics and income level

Across all three provinces, household income was not significantly associated with improved LDR perceptions. Saskatchewan showed the largest positive estimated odds ratio (OR = 1.026), although the magnitude of the association was minimal and statistically non-significant.

## 4 Discussion

Table 7 provides a summary of the major findings of this study discussed in each section.

The findings suggest that participants generally expressed relatively low levels of concern regarding LDR even before exposure to the educational materials. Following participation in the ECS, participants reported more favorable perceptions of LDR. Given the study design, these findings should be interpreted as immediate self-reported shifts in perception rather than evidence that any specific component of the ECS caused the observed changes.

One possible explanation for these findings is that many members of the public have limited prior exposure to information regarding radiation science and often rely on intuitive judgments when evaluating technological risks. Educational materials that provide contextual information, examples, and comparisons may help participants interpret radiation-related concepts within a broader framework, potentially reducing uncertainty and increasing confidence in their understanding of the subject.

The results are broadly consistent with previous literature indicating that risk perceptions are influenced not only by technical information but also by communication format, familiarity, trust in institutions, and the accessibility of scientific information. Radiation risks are often perceived differently from other hazards because they are invisible, difficult to personally evaluate, and frequently associated with nuclear accidents or other high-profile events. Accordingly, favorable shifts in self-reported perceptions may occur following educational communication activities even when the underlying scientific information remains unchanged.

Differences observed between the two infographic formats may also provide useful insights for science communication. Infographic 1 relied more heavily on analogies and comparisons to familiar experiences, whereas Infographic 2 emphasized factual and contextual information. Although the study was not designed to formally compare communication mechanisms, the observed patterns suggest that different communication approaches may be associated with different participant responses. Future research could examine these mechanisms more directly using experimental designs specifically developed to compare communication strategies.

At the same time, several limitations should be considered when interpreting the findings. The study employed a single-group pretest-posttest design and did not include a separate control group. Consequently, the results should not be interpreted as demonstrating definitive causal effects of the ECS or any of its individual components. Alternative explanations, including repeated measurement effects, focus group interactions, social desirability bias, and broader discussion dynamics, may also have contributed to observed changes in participant responses.

An additional limitation concerns the communication materials themselves. The infographics were developed using scientific and institutional sources and reviewed by subject-matter experts; however, different audiences may interpret specific examples, analogies, or framing approaches differently. Future studies could compare alternative educational materials presenting different communication styles or scientific perspectives regarding low-dose radiation in order to better understand how message framing influences public perceptions.

From a policy perspective, these findings contribute to ongoing discussions regarding risk communication, public trust, and science-based decision making. Public understanding of radiation-related issues is increasingly important in contexts involving nuclear energy, medical applications, environmental policy, and emerging technologies. The findings suggest that ECS incorporating infographic-based materials may be associated with short-term favorable shifts in self-reported perceptions among some participants. Understanding how educational communication sessions incorporating infographic-based materials relate to participants’ perceptions could assist policymakers, educators, regulators, and industry stakeholders in developing public communication strategies.

Future research should incorporate larger samples, preregistered analytical plans, randomized control groups, and longitudinal follow-up measurements. Such approaches would allow stronger conclusions regarding the persistence of attitude changes and the causal impact of different communication interventions.

### 4.1 Study limitations

Several limitations should be considered when interpreting these findings.

First, the study employed a single-group pretest-posttest design and did not include a separate control group. Consequently, the observed changes cannot be interpreted as definitive evidence that any individual component of the ECS caused the observed differences in participant responses.

Second, the study was exploratory in nature and was not preregistered. Future research would benefit from preregistered hypotheses and analytical plans to strengthen methodological transparency.

Third, the sample size limits statistical power and restricts the ability to detect small demographic effects. Given the relatively small provincial sample sizes and the inclusion of five predictors in each model, the regression estimates may also be sensitive to sample composition and should therefore be interpreted as exploratory. Although the age coefficient in the combined provincial analysis was statistically significant (OR = 0.781, *p* = 0.043), the overall combined regression model did not reach conventional statistical significance (Omnibus *p* = 0.243), and no individual demographic predictor reached statistical significance within the provincial models. Consequently, the observed demographic patterns should be interpreted cautiously as exploratory findings pending replication in larger samples.

Fourth, focus group interactions, repeated measurement effects, and social desirability effects may have influenced participant responses independently of the infographic content.

Finally, the communication materials examined in this study represent only two approaches to communicating information about low-dose radiation. Alternative communication strategies, message framing approaches, and educational formats may produce different outcomes.

### 4.2 Future research

Future research could build upon the findings of this exploratory study in several ways. First, the inclusion of randomized control groups would strengthen the ability to distinguish the effects of specific components of the ECS from other potential influences. Larger sample sizes would also improve statistical power and allow for more robust examination of demographic differences across age, gender, education, income, and regional contexts.

Future studies may also benefit from evaluating alternative approaches to measuring perceptions of low-dose radiation, including validated multi-item scales, ordinal models, and other operationalizations that capture a broader range of participant attitudes. In addition, researchers could compare different communication strategies, such as natural analogies, technical explanations, regulatory information, and alternative message-framing approaches, to better understand which forms of communication are most effective in different contexts.

Longitudinal research would be particularly valuable in determining whether observed changes in perceptions persist over time or diminish following the intervention. Replication across additional provinces, countries, and occupational groups could further improve understanding of how social, cultural, and regional factors influence responses to low-dose radiation communication. Finally, future studies could examine how different presentations of scientific uncertainty and competing low-dose radiation risk models influence public trust, risk perception, and policy preferences.

## 5 Conclusion

The findings indicate that participants generally reported more favorable perceptions of low-dose radiation following participation in the Educational Communication Sessions, although a small minority reported less favorable post-session perceptions. Participants also reported relatively high levels of trust in scientists following the sessions. However, the study design does not permit causal attribution of these changes to the infographics or any individual component of the ECS.

Overall, this exploratory study provides preliminary evidence of immediate self-reported shifts in LDR perceptions within a non-representative focus-group sample. The findings should therefore be interpreted cautiously and do not establish that the ECS caused the observed changes. Larger studies incorporating control groups, longitudinal follow-up, and alternative communication approaches are needed to assess the robustness and persistence of these patterns.

## Data Availability

The raw data supporting the conclusions of this article will be made available by the authors, without undue reservation.
