# Supplementary material for: Dynamics of Canadian perceptions of low-dose radiation following science-based educational communication sessions
Source: Front Public Health. 2026 Oct 9;14:1976699. doi: 10.3389/fpubh.2026.1976699 (PMC13690554; doi:10.3389/fpubh.2026.1976699)
Supplement: Supplementary file 1 [file Supplementary_file_1.pdf]

Supplementary Material – Appendices A–D

Appendix A: Infographic 1

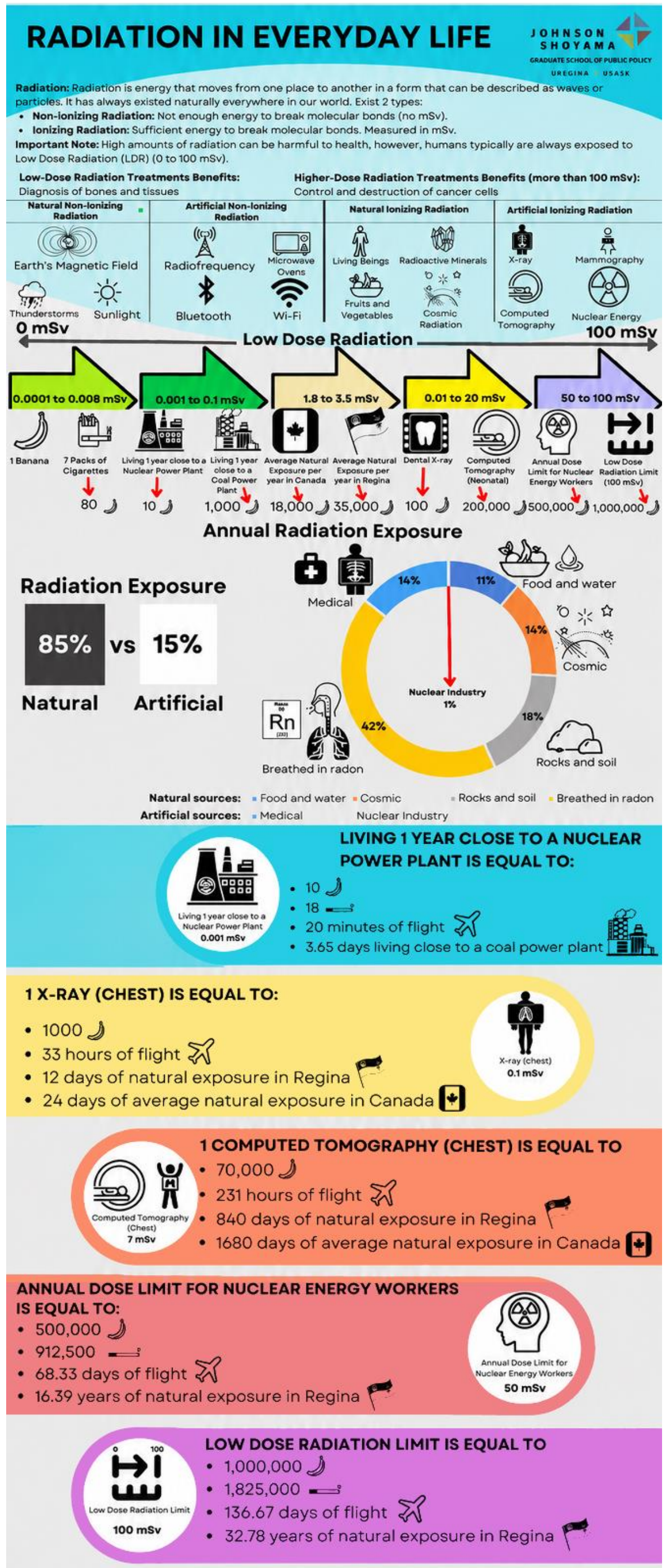

This infographic was originally published in (Sahagún-Aguilar and Hurlbert 2025) and is reused here by the authors under the CC BY 4.0 license.

Appendix B: Infographic 2

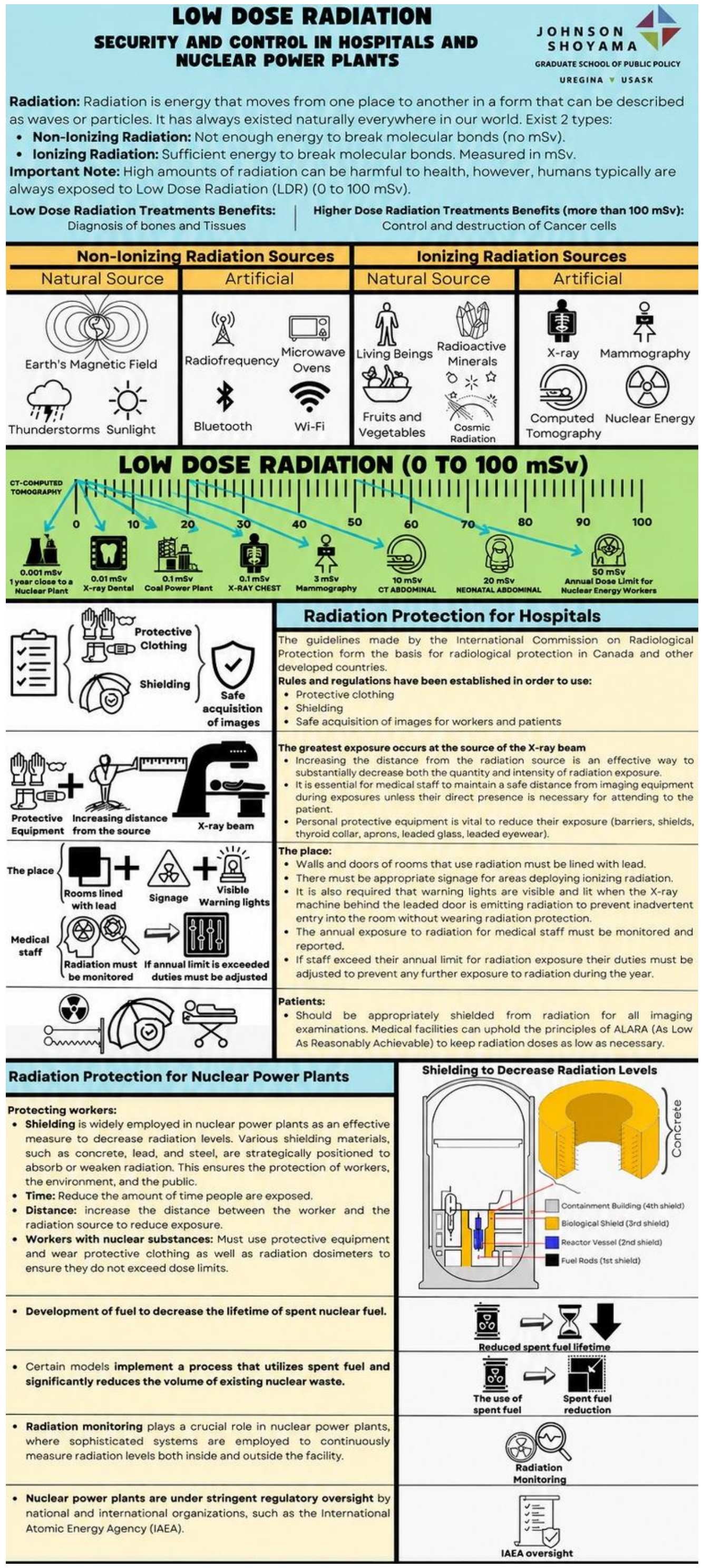

This infographic was originally published in (Sahagún-Aguilar and Hurlbert 2025) and is reused here by the authors under the CC BY 4.0 license.

Appendix C

Creation of the binary outcome variable. The dependent variable represented whether participants reported an improved LDR perception following participation in the Educational Communication Session. Participants were coded as 1 when their post-session LDR perception score was more favorable than their pre-session score and 0 otherwise.

Appendix C.1: SPSS Binary Logistic Regression Calculations in Saskatchewan

Omnibus Test

The overall binary logistic regression model was not statistically significant ($\chi^2 = 5.466$, df = 5, p = 0.362), indicating that the demographic variables included in the model did not significantly explain which participants reported improved LDR perceptions following participation in the ECS.

Model Summary and Goodness-of-Fit Diagnostics
The model was estimated using 43 participants, with 3 cases excluded because of missing data. The −2 Log Likelihood was 50.152. The Cox & Snell R^2 was 0.119 and the Nagelkerke R^2 was 0.164. The Hosmer–Lemeshow test was not statistically significant ($\chi^2 = 6.087$, df = 8, p = .638).

Variables in the Equation

		B	S.E.	Wald	df	Sig.	Exp(B)	95% C.I.for EXP(B)	
								Lower	Upper
Step 1[a]	Infographic	.662	.726	.832	1	.362	1.938	.467	8.040
	Gender	-.345	.640	.291	1	.590	.708	.202	2.482
	Age	-.588	.340	2.991	1	.084	.555	.285	1.082
	Education	-.128	.236	.295	1	.587	.880	.554	1.397
	Household_Income	.026	.225	.013	1	.909	1.026	.661	1.593
	Constant	1.263	2.610	.234	1	.629	3.535		

a. Variable(s) entered on step 1: Infographic, Gender, Age, Education, Household_Income.

1 Interpretation

Infographic. The infographic format was not significantly associated with improved LDR perceptions (OR = 1.938, $p = .362$). Although the estimated odds ratio was greater than one, the association should be interpreted only as an exploratory directional tendency.

Gender. No statistically significant association was observed between gender and improved LDR perceptions (OR = 0.708, $p = .590$).

Age. Older participants showed lower estimated odds of reporting improved LDR perceptions (OR = 0.555, $p = .084$). Although this association did not reach statistical significance, it represented the strongest directional tendency observed in Saskatchewan and may warrant further investigation in larger samples.

Education. Education was not significantly associated with improved LDR perceptions (OR = 0.880, $p = .587$).

Household Income. Household income showed no meaningful association with improved LDR perceptions (OR = 1.026, $p = .909$).

Appendix C.2: SPSS Binary Logistic Regression Calculations in Alberta

Omnibus Test

The overall binary logistic regression model was statistically significant ($\chi^2 = 11.675$, df = 5, $p = .040$), indicating that the set of demographic variables jointly contributed to explaining which participants reported improved LDR perceptions following participation in the Educational Communication Session.

Model Summary and Goodness-of-Fit Diagnostics

The model was estimated using 54 participants, with no cases excluded because of missing data. The −2 Log Likelihood was 57.069. The Cox & Snell R^2 was 0.194 and the Nagelkerke R^2 was 0.270. The Hosmer–Lemeshow test was not statistically significant ($\chi^2 = 6.066$, df = 8, $p = .640$).

Variables in the Equation

		B	S.E.	Wald	df	Sig.	Exp(B)	95% C.I.for EXP(B)	
								Lower	Upper
Step 1[a]	Infographic	-.534	.688	.604	1	.437	.586	.152	2.256
	Gender	1.062	.665	2.549	1	.110	2.891	.785	10.646
	Age	-.222	.224	.982	1	.322	.801	.516	1.242
	Education	-.061	.277	.048	1	.826	.941	.547	1.619
	Household_Income	-.052	.068	.573	1	.449	.950	.831	1.085
	Constant	-.091	1.916	.002	1	.962	.913		

a. Variable(s) entered on step 1: Infographic, Gender, Age, Education, Household_Income.

Interpretation

Infographic. No statistically significant association was observed between infographic format and improved LDR perceptions (OR = 0.586, p = .437).

Gender. Gender showed the largest estimated association with improved LDR perceptions (OR = 2.891, p = .110). Although this coefficient did not reach statistical significance, it represented the strongest directional tendency within the Alberta model.

Age. Age was not significantly associated with improved LDR perceptions (OR = 0.801, p = .322).

Education. Education showed no statistically significant association with improved LDR perceptions (OR = 0.941, p = .826).

Household Income. Household income was not significantly associated with improved LDR perceptions (OR = 0.950, p = .449).

Appendix C.3: SPSS Binary Logistic Regression Calculations in Ontario

Omnibus Test

The overall binary logistic regression model was not statistically significant ($\chi^2 = 2.959$, df = 5, p = .706), indicating that the demographic variables did not significantly explain which participants reported improved LDR perceptions following participation in the Educational Communication Session.

Model Summary and Goodness-of-Fit Diagnostics

The model was estimated using 50 participants. The −2 Log Likelihood was 65.070. The Cox & Snell R^2 was 0.057 and the Nagelkerke R^2 was 0.077. The Hosmer–Lemeshow test was not statistically significant ($\chi^2 = 15.377$, df = 8, p = .052).

Variables in the Equation

		B	S.E.	Wald	df	Sig.	Exp(B)	95% C.I.for EXP(B)	
								Lower	Upper
Step 1[a]	Infographic	-.151	.607	.062	1	.804	.860	.262	2.827
	Gender	-.633	.607	1.089	1	.297	.531	.162	1.743
	Age	-.136	.189	.516	1	.472	.873	.603	1.265
	Education	.198	.285	.480	1	.489	1.219	.696	2.132
	Household_Income	.005	.012	.170	1	.680	1.005	.982	1.028
	Constant	.236	2.054	.013	1	.908	1.266		

a. Variable(s) entered on step 1: Infographic, Gender, Age, Education, Household_Income.

Interpretation

Infographic. No statistically significant association was observed between infographic format and improved LDR perceptions (OR = 0.860, p = .804).

Gender. Gender was not significantly associated with improved LDR perceptions (OR = 0.531, p = .297).

Age. Older participants showed slightly lower estimated odds of reporting improved LDR perceptions (OR = 0.873, p = .472), although the association was not statistically significant.

Education. Education showed no statistically significant association with improved LDR perceptions (OR = 1.219, p = .489).

Household Income. Household income was not significantly associated with improved LDR perceptions (OR = 1.005, p = .680).

Appendix C.4: SPSS Binary Logistic Regression Calculations Combining Saskatchewan, Alberta, and Ontario

Omnibus Test

The overall binary logistic regression model combining participants from Saskatchewan, Alberta, and Ontario was **not statistically significant** ($\chi^2 = 6.708$, df = 5, p = .243), indicating that the set of independent variables did not significantly explain which participants reported improved LDR perceptions following participation in the Educational Communication Session.

Model Summary and Goodness-of-Fit Diagnostics

The model included 147 participants, with 3 cases excluded because of missing data. The −2 Log Likelihood was 186.605. The Cox & Snell R^2 was 0.045 and the Nagelkerke R^2 was 0.061. The Hosmer–Lemeshow test was not statistically significant ($\chi^2 = 5.855$, df = 8, p = .663).

Variables in the Equation

		B	S.E.	Wald	df	Sig.	Exp(B)	95% C.I.for EXP(B)	
								Lower	Upper
Step 1[a]	Infographic	.058	.352	.027	1	.869	1.060	.531	2.114
	Gender	.082	.336	.060	1	.807	1.086	.562	2.098
	Age	-.247	.122	4.109	1	.043	.781	.615	.992
	Education	-.058	.132	.191	1	.662	.944	.728	1.223
	Household_Income	-.010	.008	1.299	1	.254	.990	.974	1.007
	Constant	.498	1.097	.206	1	.650	1.645		

a. Variable(s) entered on step 1: Infographic, Gender, Age, Education, Household_Income.

Interpretation

Infographic. No statistically significant association was observed between infographic format and improved LDR perceptions (OR = 1.060, p = .869). The estimated odds were approximately 6.0% higher for the non-reference infographic category relative to Infographic 1, although this difference was not statistically significant.

Gender. Gender was not significantly associated with improved LDR perceptions (OR = 1.086, p = .807). The estimated odds were approximately 8.6% higher for males relative to the female reference category, although this association was not statistically significant.

Age. Increasing age was associated with lower estimated odds of reporting improved LDR perceptions (OR = 0.781, p = .043). Each one-category increase in age was associated with an estimated 21.9% decrease in the odds of reporting improved LDR perceptions. Although the age coefficient reached statistical significance at the conventional 0.05 threshold, the overall regression model was not statistically significant (Omnibus p = .243). Therefore, this finding should be interpreted cautiously as an exploratory association rather than as confirmatory evidence of an age effect.

Education. Education was not significantly associated with improved LDR perceptions (OR = 0.944, p = .662). Each one-category increase in education was associated with an estimated 5.6% decrease in the odds of reporting improved LDR perceptions, although this association was not statistically significant.

Household Income. Household income was not significantly associated with improved LDR perceptions (OR = 0.990, p = .254). Each one-category increase in household income was associated with an estimated 1.0% decrease in the odds of reporting improved LDR perceptions, although this association was not statistically significant.

APPENDIX D: Survey

- Survey begins -

1. What is your age?

   [number]

2. What is your gender?

   Man

   Woman

   Non-binary

   Prefer not to say

3. What is your income level? (use statistics Canada breakdowns)
4. What is your highest level of education (“”)
5. Statistics Canada ethnicity identification

Background questions:

A1. Overall, how would you rate your personal knowledge about radiation. Would you say that it is …:

1. Very good
2. Good
3. Moderate
4. Poor
5. Very poor
98. (Don't know)
99. (Refused)

A2. Do you have personal experience working in a job that involves the use of radioactivity (nuclear power plant, industry or hospital using radioactive sources, natural radioactivity in ores and other materials)?

A3. On a scale of 1-5 (with 1 'not at all' to 5 'very' and 3 'neutral'), how ____ do you feel about deploying small modular nuclear reactors (SMRs) as a climate change solution?

(Substitute sad, anxious, angry, excited)

FOCUS GROUP QUESTIONS:

1. On a scale from 1 to 5 where 1 is safe and 5 is dangerous, what is your perception when you hear the term Low Dose Radiation? (with 1 'safe' to 5 'dangerous' and 3 'neutral')
2. Have you heard the phrase Low Dose Radiation before? What comes to mind when you hear that phrase Low Dose Radiation?
3. Where is Low Dose Radiation found?
4. What concerns do you have about Low Dose Radiation?
5. What are the potential benefits of Low Dose Radiation?

PRESENTATION OF INFOGRAPHIC/ BY VIDEO

6. How do you feel knowing that low dose radiation is found everywhere? Including that it is a natural element in our body, the food we eat, and has always existed.
7. What do you think about having cheaper and wider access to medical services using LDR?

8. Knowing that a scientist is conducting this research, does having access to all the scientific sources of information make you feel safer? Why or why not?
9. Only for infographic 1 participants. Did the analogies based in nature help you understand better Low Dose Radiation? Why or why not?

9. Only for infographic 2 participants. Did hearing about the security and control of radiation in hospitals and nuclear power plants make you feel safer when thinking about radiation? Why or why not?

10. After the infographic, did anything change about your personal perception about Low Dose Radiation? Why and why not?
11. Thinking about the benefits of Low Dose Radiation outweigh the risks? Why or why not?

EXIT QUESTIONS

12. On a scale from 1 to 5 where 1 is safe and 5 is dangerous, what is your perception when you hear the term Low Dose Radiation? (with 1 'safe' to 5 'dangerous' and 3 'neutral')

13. On balance, the benefits of radiation outweigh the risks?

    1. Strongly Agree
    2. Agree
    3. Undecided
    4. Disagree Strongly
    5. Disagree

14. On a scale of 1-5 (with 1 'not at all' to 5 'very' and 3 'neutral'), how ____ do you feel about deploying small modular nuclear reactors (SMRs) as a climate change solution?
